# Supplementary material for: A Lifelong Impact on Endometriosis: Pathophysiology and Pharmacological Treatment
Source: Int J Mol Sci. 2023 Apr 19;24(8):7503. doi: 10.3390/ijms24087503 (PMC10139092; doi:10.3390/ijms24087503)
Supplement: Supplementary file 1 [file ijms-24-07503-s001.zip › ijms-2234683-supplementary.pdf]

International Journal of *Molecular Sciences*

MDPI

Endometriotic lesions		<1cm	1-3cm	>3cm
Peritoneum	Superficial	1	2	4
	Deep	2	4	6
Ovary, right	Superficial	1	2	4
	Deep	4	16	20
Ovary, left	Superficial	1	2	4
	Deep	4	16	20
Cul-de-sac obliteration	Partial		Complete	
	4		40	
**Adhesions**		**<1/3 Enclosure**	**1/3-2/3 Enclosure**	**>2/3 Enclosure**
Ovary, right	Filmy	1	2	4
	Dense	4	8	16
Ovary, left	Filmy	1	2	4
	Dense	4*	8*	16
Tube, right	Filmy	1	2	4
	Dense	4*	8*	16
Tube, left	Filmy	1	2	4
	Dense	4	8	16

rASRM stage	Total score
Stage I (Minimal)	1-5
Stage II (Mild)	6-15
Stage III (Moderate)	16-40
Stage IV (Severe)	>40

* If the fimbriated end of the fallopian tube is completely enclosed, change the point assignment to 16.

Figure S1. The severity of pelvic endometriosis. After surgical intervention, the severity of pelvic endometriosis can be determined using the revised scoring system of the American Society for Reproductive Medicine (ASRM).
